# Structural Insights into *N*^6^-methyladenosine (m^6^A) Modification in the Transcriptome

**DOI:** 10.1016/j.gpb.2018.03.001

**Published:** 2018-04-27

**Authors:** Jinbo Huang, Ping Yin

**Affiliations:** National Key Laboratory of Crop Genetic Improvement and National Center of Plant Gene Research, Huazhong Agricultural University, Wuhan 430070, China

**Keywords:** Epitranscriptomics, M^6^A modification, Writer, Reader, Eraser

## Abstract

More than 100 types of chemical modifications in RNA have been well documented. Recently, several modifications, such as *N*^6^-methyladenosine (m^6^A), have been detected in mRNA, opening the window into the realm of **epitranscriptomics**. The **m^6^A modification** is the most abundant modification in mRNA and non-coding RNA (ncRNA). At the molecular level, m^6^A affects almost all aspects of mRNA metabolism, including splicing, translation, and stability, as well as microRNA (miRNA) maturation, playing essential roles in a range of cellular processes. The m^6^A modification is regulated by three classes of proteins generally referred to as the “**writer**” (adenosine methyltransferase), “**eraser**” (m^6^A demethylating enzyme), and “**reader**” (m^6^A-binding protein). The m^6^A modification is reversibly installed and removed by writers and erasers, respectively. Readers, which are members of the YT521-B homology (YTH) family proteins, selectively bind to RNA and affect its fate in an m^6^A-dependent manner. In this review, we summarize the structures of the functional proteins that modulate the m^6^A modification, and provide our insights into the m^6^A-mediated gene regulation.

## Introduction

RNA plays a significant role in the life cycle. Since the 1950s, more than 100 types of chemical modifications have been described in RNA, particularly in rRNA and tRNA [1]. Benefitting from the recent technological advances, several modifications have been detected in mRNA [2], such as *N*^6^-methyladenosine (m^6^A) [3], [4], 5-methylcytosine (m^5^C) [5], [6], *N*^1^-methyladenosine (m^1^A) [7], [8], pseudouridine (Ψ) [9], [10], and inosine (I) [2], [11]. These modifications reveal a widespread and sparse landscape of epitranscriptomics, which is involved in a variety of physiological processes [2], [12], [13], [14].

The m^6^A modification, a rising star in the epitranscriptomics field, is the most pervasive modification found in transcripts [13], [15]. First discovered in 1974 in mRNA from cancer cells [16], m^6^A was subsequently detected in large quantities in various viruses [17], [18], [19], yeast [20], [21], [22], *Arabidopsis* [18], [23], [24], *Drosophila* [25], [26], [27], mice [28], [29], [30], and humans [3], [4], [12], [31], [32], [33]. The m^6^A modification is involved in almost all aspects of mRNA metabolism, including splicing [26], [34], [35], stability [35], [36], [37], [38], and translation efficiency [36], [39], [40], [41], as well as long ncRNA (lncRNA)-mediated transcriptional repression [42] and miRNA maturation [43]. Several excellent review articles have summarized the biological functions of m^6^A, which include cell differentiation, immune homeostasis, mitosis, obesity, cancer, and the maintenance of biological rhythm, among others [12], [13], [14], [31], [32], [33], [44], [45], [46], [47], [48], [49].

The m^6^A modification is usually found within the conserved motif containing RRACH (where A is methylated; R = purine, and H = A, C or U) [3], [4], [50], [51]. The m^6^A modification process is regulated by three types of proteins commonly termed the “writer” (adenosine methyltransferase), “eraser” (m^6^A-demethylating enzyme), and “reader” (m^6^A-binding protein) [12], [13], [15], [49]. This modification is reversibly installed and removed by the writer proteins [31], [52], [53], [54], [55] and eraser proteins [28], [56], respectively [12], [13], [14]. Conceivably, m^6^A is also specifically recognized by m^6^A reader proteins that contain the YT521-B homology (YTH) domain [3], [12], [13], [14], [49]. Recent studies on m^6^A have broadened our knowledge of epitranscriptomics.

On this review, we focus on the components of the m^6^A writer complex and the principles of the reversible process from the perspective of structural biology, aimed to provide new insights into the molecular mechanisms underlying the m^6^A modification. We hope that the structural information could potentially contribute to the development of therapeutic agents.

## Insights into the structure of the METTL3–METTL14 complex

**Figure 1:**
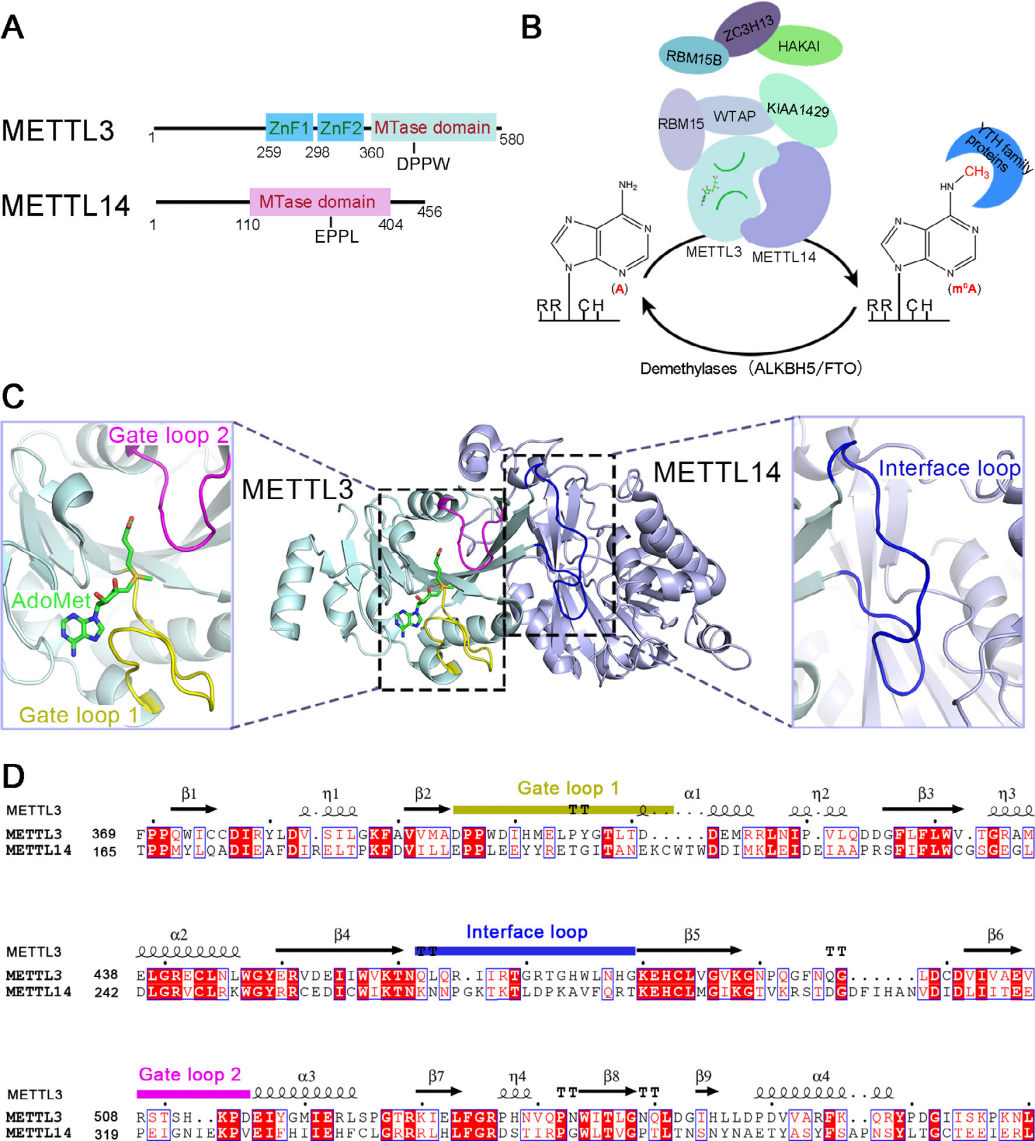
**Structural insights into the METTL3–METTL14 complex as an m^6^A writer** **A.** Schematic illustration of the METTL3 (GenBank accession: NP_062826.2) and METTL14 (GenBank accession: NP_066012.1). The ZnF domains (ZnF1: AA residues 259–298; ZnF2: AA residues 299–336) and the MTase domain (AA residues 360–580) in METTL3 are colored in cyan and light blue, respectively. The MTase domain (AA residues 110–404) in METTL14 is colored in light purple. The DPPW and EPPL are the conserved catalytic motif in METTL3 and METTL14, respectively. **B.** The reversible m^6^A modification in mRNA is installed, removed, and recognized by m^6^A writers, erasers, and readers, respectively. The A in RRACH is methylated by the m^6^A writer complex, which comprises several components, including METTL3, METTL14, WTAP, KIAA1429, RBM15/RBM15B, ZC3H13, and HAKAI. The methylated RNA can be demethylated by erasers, ALKBH5 and FTO. Also, m^6^A is specifically recognized by m^6^A readers, which are members of the YTH family proteins. **C.** Overall structure of the AdoMet-bound heterodimer of METTL3–METTL14 (PDB ID: 5IL1) and close-up view of the gate loop 1 (purple), gate loop 2 (yellow), and interface loop (blue). **D.** Sequence alignment of the MTDs of human METTL3 and METTL14 proteins. The secondary structure of METTL3 is shown on the top with the detailed AA sequences shown below. The α-helices, β-strands, and strict β-turns are displayed as squiggles, arrows, and TT letters, respectively. Identical AA residues between METTL3 and METTL14 proteins are shown in white letters with a red background, and similar AA residues are shown in red letters. AdoMet, *S*-adenosylmethionine; ALKBH5, AlkB homolog 5; FTO, fat mass and obesity-associated protein; HAKAI, E3 ubiquitin-protein ligase Hakai; KIAA1429, vir-like m^6^A methyltransferase associated protein; METTL3, methyltransferase-like 3; MTase, methyltransferase; RBM15/15B, RNA binding motif protein 15/15B; WTAP, Wilms’ tumor 1-associating protein; YTH, YT521-B homology; ZC3H13, zinc finger CCCH domain-containing protein 13; ZnF, zinc finger domain.

The enzyme that catalyzes the m^6^A modification was first isolated in the 1990s [57], [58]. Rottman and colleagues identified at least two separate protein factors, methyltransferase component A (MT-A) and MT-B, both of which are able to install m^6^A in mRNA. One component of the MT-A complex, the *S*-adenosylmethionine (SAM or AdoMet)-binding site on a 70-kDa subunit (MT-A70), was characterized as a key subunit of methyltransferase [58]. Lately, MT-A70 was assigned as methyltransferase-like 3 (METTL3) by the Human Genome Organization (HUGO) Gene Nomenclature Committee [59]. In all the organisms that have been examined so far, an induced experimental deficiency of *METTL3* or its homologs leads to apoptosis [37], defects in gametogenesis [60], and embryonic lethality [23]. In 2014, several groups independently revealed that METTL3 interacts with METTL14 to form a stable complex [52], [53], [54], [55], which deposits m^6^A on mRNAs. According to phylogenetic analyses of the MT-A70 family, METTL14 is a homolog of METTL3 [61], [62]. Both of these homologs possess a methyltransferase domain (MTase domain or MTD) (Figure 1A), a consensus fold in the methyltransferase family [61], [62]. Knockdown of *METTL3* and *METTL14* dramatically reduces m^6^A levels in mammalian cells [52], [53]. The METTL3–METTL14 complex exhibits significantly-increased methyltransferase activity *in vitro* compared to each individual protein alone [52], [53], [63], [64]. Thus, both METTL3 and METTL14 are core components of the m^6^A writer complex (Figure 1B and Table 1).

**Table 1:** **m^6^A writer proteins and homologs across different species**

***Homo sapiens***	***Mus musculus***	***Drosophila melanogaster***	***Saccharomyces cerevisiae***	***Arabidopsis thaliana***	***Oryza sativa***
METTL3	Mettl3	IME4	Ime4	AtMTA	MT-A70-like
METTL14	Mettl14	dMettl14(CG7818)	Kar4	AtMTB	XP_015614540
WTAP	Wtap	Fl(2)d	Mum2	FIP37	FIP37
KIAA1429 (VIRMA)	Virilizer	Virilizer	Unknown	Virilizer	Unknown
RBM15	Rbm15	Spenito	Unknown	Unknown	Unknown
RBM15B	Rbm15B	Unknown	Unknown	Unknown	Unknown
HAKAI	Hakai	Hakai	Unknown	Hakai	Hakai
METTL16	Mettl16	Unknown	Unknown	Unknown	Unknown
Unknown	Unknown	Unknown	Slz1	Unknown	Unknown

*Note*: AtMTA, mRNA adenosine methylase in *Arabidopsis thaliana*; AtMTB, ortholog of human METTL14 in *Arabidopsis thaliana*; FIP37, FKBP12-interacting protein of 37 kDa; Fl(2)d, female-lethal (2)d; HAKAI, E3 ubiquitin-protein ligase Hakai; Ime4, inducer of meiosis 4; Kar4, karyogamy protein KAR4; KIAA1429 (VIRMA), vir-like m^6^A methyltransferase associated protein, or Virilizer homolog; METTL3, methyltransferase-like 3; MT-A70, the *S*-adenosylmethionine-binding site on a 70-kDa subunit; Mum2, muddled meiosis protein 2; RBM15/15B, RNA binding motif protein 15/15B; Slz1, sporulation-specific with a leucine zipper motif protein 1; WTAP, Wilms’ tumor 1-associating protein.

Recently, we and two other groups independently determined the crystal structures of the MTDs of the METTL3–METTL14 complex in the presence or absence of ligands [63], [64], [65] (Figure 1C and Table 2). The overall structure of the binary MTD complex resembles a flying butterfly (Figure 1C) [63]. In the asymmetric unit, the MTDs of METTL3 and METTL14 form a 1:1 heterodimer, consistent with observations in solution [64]. The structures of both MTDs are nearly identical. The overall structure of the METTL3 MTD adopts a typical α-β- α sandwich fold, a classical Rossman fold-like domain [62], [63], [64], [65]. This domain contains eight β sheets flanked by four α-helices and three 3_10_ helices. The interface between METTL3 and METTL14 is approximately 2500 Å^2^ [63]. More than 20 amino acid (AA) residues form extensive interactions with each other in various ways, including salt bridges, water-mediated hydrogen bonding, and hydrophobic interactions [63], [64]. Thus, the heterocomplex is difficult to disrupt. These observations explain why both domains are imperative for proper folding, stability, and activity [63], [64], [65].

**Table 2:** **Structural information of m^6^A writers/erasers/readers**

**Category**	**Protein name**	**Species**	**PDB ID**	**Resolution (Å)**	**Substrate/ligand**	**Ref.**
Writer	METTL3–METTL14 complex	*Homo sapiens*	5IL0/5IL1/5IL2	1.88/1.71/1.61	Apo/AdoMet/AdoHcy	[63]
		*Homo sapiens*	5K7M/5K7U/5K7W	1.65/1.7/1.65	Apo/AdoMet/AdoHcy	[64]
		*Homo sapiens*	5L6E/5L6D	1.9/1.85	AdoMet/AdoHcy	[65]

Eraser	ALKBH5	*Homo sapiens*	4NJ4	2.02	IOX3	[78]
		*Danio rerio*	4NPL/4NPM	1.65/1.8	α-KG/SIN	[86]
		*Homo sapiens*	4NRM/4NRO/4NRP/4NRQ/4O7X	2.17/2.3/1.8/2.5/1.78	CIT and ACT/α-KG/NOG/PD2/Apo	[95]
		*Homo sapiens*	4O61/4OCT	1.9/1.65	CIT/α-KG	[94]
	
	FTO	*Homo sapiens*	3LFM	2.5	1-mer 3-meT and NOG	[77]
		*Homo sapiens*	4IDZ/4IE0/4IE4/4IE5/4IE6/4IE7	2.46/2.53/2.5/1.95/2.5/2.6	NOG/PD2/8XQ/MD6/IOX3/RHN and CIT	[80]
		*Homo sapiens*	4QKN	2.2	MA and NOG	[97]
		*Homo sapiens*	4ZS2/4ZS3	2.16/2.45	α-KG and FL1/α-KG and FL4	[98]

Reader	YTHDC1	*Homo sapiens*	4R3H/4R3I	1.9/1.8	Apo/5-mer m6A RNA	[105]
	RnYTHDC1	*Rattus norvegicus*	2MTV	Solution structure	6-mer m6A RNA	[114]
	YTHDF1	*Homo sapiens*	4RCI/4RCJ	1.97/1.6	Apo/5-mer m6A RNA/	[117]
	YTHDF2	*Homo sapiens*	4WQN	2.12	Apo	[115]
	YTHDF2	*Homo sapiens*	4RDO/4RDN	2.15/2.1	Apo/1-mer m6A	[116]
	ScPho92	*Saccharomyces cerevisiae*	4RCM	1.8	1-mer m6A	[117]
	ZrMRB1	*Zygosaccharomyces rouxii*	4U8T	2.7	7-mer m6A RNA	[113]

*Note*: AdoMet, *S*-adenosylmethionine; AdoHcy, *S*-adenosyl-L-homocysteine; IOX3, (1-chloro-4-hydroxyisoquinoline3-carbonyl) glycine; α-KG, alpha-ketoglutaric acid; SIN, succinate acid; CIT, citrate; ACT, acetate; NOG, N-oxalylglycine; PD2, pyridine-2,4-dicarboxylate; 8XQ, 8-hydroxyquinolines; MD6, 2-(3-hydroxypicolinamido) acetic acid; RHN, rhein; MA, meclofenamic acid; FL1, fluorescein derivative 1; FL4, fluorescein derivative 4.

Intriguingly, three loops are present in METTL3 [63] (Figure 1C and D), namely, gate loop 1 (AA residues 395–410), interface loop (AA residues 462–479), and gate loop 2 (AA residues 507–515). The METTL3 structure superimposes well on the METTL14 structure, except for these three loops, suggesting that the distinct conformations may confer differential functions [31], [63].

As shown in the AdoMet-bound complex, the AdoMet molecule is positioned adjacent to the gate loop 1 and the gate loop 2 (Figure 1C) and is stabilized in the pocket by numerous favorable AA residues. Mutations of these residues significantly lead to a decrease in methylation activity [63]. Moreover, the highly conserved catalytic motif, DPPW (AA residues 395–398), is located in the gate loop 1. In this motif, the aspartic D395 is located close to the methyl moiety of AdoMet. This proximity provides support for the methyltransferase to directly transfer the methyl group from AdoMet to the *N*^6^ position of the adenine residue of RNA with a conformational inversion in an S_N_2 reaction [31], [63], [66]. Thus, both gate loop 1 and gate loop 2 contribute to the formation of catalytic center for coordinating AdoMet.

Furthermore, compared with the *S*-adenosylhomocysteine (AdoHcy)-bound structure, gate loop 1 is turned outward in the AdoMet-bound structure in METTL3. Likewise, gate loop 2 experiences a meaningful conformational change upon binding to AdoMet or AdoHcy, leading to the closure of the binding pocket [63]. These rearrangements in gate loop 1 and gate loop 2 of METTL3 resemble those in loop 1 and loop 2 of *N*^6^-MTase TaqI (M.TaqI) when interacting with its DNA substrate, suggesting that the gate loop 1 and gate loop 2 play critical roles in RNA substrate recognition [63], [67].

The interface loop of METTL3 forms a groove together with the N-terminal α-helical motif and the long linker that connects β 5 and β 6 of METTL14 [63]. The structure of the complex shows that the groove primarily mediates the interactions between METTL3 and METTL14. The electrostatic potential analysis of the AdoMet-bound structure indicates that a large number of positively-charged AA residues on the surface of the groove neighboring the AdoMet ligand [63], [64]. Mutations of the interface loop result in the lower RNA-binding activity and a subsequent decrease in the methylation activity [63], [64], but do not affect the AdoMet-binding ability [31], [63]. These findings indicate that the interface loop contributes to protein–protein and protein–RNA interactions.

Together, gate loop 1, gate loop 2, and the interface loop play important roles in ligand binding, RNA recognition, and the mutual interactions between the two MTDs. Although the binary structure of the MTDs of METTL3–METTL14 has been determined, we do not yet completely understand the molecular mechanisms of how writers recognize the consensus RNA sequence, due to the lack of a structure of the protein–RNA complex. Further investigations are required to confirm the highly reversible nature of the entire catalytic process and illustrate the involvement of some other factors in the writer complex.

## METTL14 is a pseudo-methyltransferase required for METTL3 activity

In 2014, He’s group and Zhao’s group independently reported that either METTL3 or METTL14 alone has methyltransferase activity, whereas the combination of METTL3 and METTL14 shows a synergistic effect on enhancing the activity remarkably compared to each isolated protein [52], [53]. In the same year, another two groups also demonstrated that METTL14 is the component of m^6^A methyltransferase complex [54], [55]. Until 2016, using bioinformatics analyses, Aravind and colleagues suggested that METTL14 would be an inactive methyltransferase because of the disruption of its active site motif [62]. Subsequently, we and another two groups provided the biochemical and structural evidence indicating that METTL14 has no detectable activity [63], [64], [65]. One possible explanation for the previous controversial observation is that METTL14 could be contaminated with endogenous METTL3 during purification procedure [64].

Although the structures of the MTDs of METTL3 and METTL14 are nearly identical, the ligand is only clearly visible in the ligand-binding pocket of MELLT3 [63], [64], [65] (Figure 1C). In contrast, neither AdoMet nor AdoHcy is observed in the ligand-binding pocket of METTL14, suggesting that METTL14 does not possess catalytic activity. Indeed, according to *in vitro* biochemical analyses, isolated METTL3, but not METTL14, exhibits detectable catalytic activity, raising the possibility that METTL14 may function as a pseudo-methyltransferase [31], [64].

The structural features of METTL14 could account for this observation. First, the ligand-binding pocket of METTL14 is smaller than that of METTL3 [64], [65]. Obvious clashes are noted between the modeled AdoMet and the ligand-binding pocket in the structure of the METTL14 MTD [64], [65]. Furthermore, two residues, N549 and Q550 in METTL3, which form hydrogen bonds with the ribose hydroxyl moiety of AdoMet, are substituted by P362 and T363 in METTL14, respectively [63]. Additionally, W398 from the conserved DPPW motif in METTL3 stabilizes the adenine substrate through stacking interactions, whereas the counterpart of the conserved motif in METTL14 is EPPL (AA residues 192–195), which lacks the aromatic residue [63], [64], [65]. Collectively, these observations indicate that METTL14 contains a pocket that is unsuitable for accommodating a methyl group donor and consequently is catalytically inactive [31], [64], [65].

Despite its lack of catalytic activity, METTL14 plays a crucial role in the activity of the writer complex. METTL14 appears to have a structural role in maintaining the integrity of the architecture of the binary complex to increase the catalytic activity [64]. Recombinant METTL3 alone shows weak catalytic activity that is dramatically stimulated when METTL3 and METTL14 form a heterodimeric complex [63], [64]. Furthermore, METTL14 increases the AdoMet binding capacity of METTL3. The METTL3–METTL14 complex binds to AdoMet with a dissociation constant of approximately 2.0 μM, showing a 24-fold stronger binding affinity than METTL3 alone (∼47.6 μM) [31], [63]. Importantly, METTL14 also contributes to the RNA interaction via the positively-charged groove (described above). The replacement of the AA residues in the groove of METTL14 results in a much weaker RNA-binding affinity and a reduced methyltransferase activity. Additionally, a recent study has reported that the RGG domain in the C-terminus of METTL14 also contributes to the RNA substrate binding for METTL3–METTL14 complex [68]. These results confirm that METTL14 is involved in the internal RNA interaction [63], [64].

In summary, METTL14 is a pseudo-methyltransferase in the complex and is indispensable for activity. It primarily serves as a platform for substrate interaction in the binary complex.

## Do other m^6^A regulators participate in the methyltransferase complex?

Several regulators in the m^6^A writer complex comprising a METTL3 protein interaction network have been identified, such as Wilms’ tumor 1-associating protein (WTAP) [23], [54], [55], KIAA1429 (vir-like m^6^A methyltransferase associated protein, or VIRMA) [55], [69], and RNA binding motif protein 15/15B (RBM15/RBM15B) [42] (Table 1 and Figure 1B). Perturbations of these factors affect cellular m^6^A levels in mammals [42], [52], [54], [55], flies [25], [26], [27], yeast [20], and plants [69].

Muddled meiosis protein 2 (Mum2) [20] and FKBP12-interacting protein of 37 kDa (FIP37) [23], the respective homologs of WTAP in yeast and plants, have been reported to interact with METTL3 homologs in these species. Downregulation of *Mum2* causes a spore formation defect in the m^6^A cellular pathway and disruption of *Fip37* leads to embryonic lethality [20], [23]. Consistent with these findings, WTAP, a splicing factor, is identified to interact with METTL3 and METTL14 in mammals by coimmunoprecipitation *in vivo* [52], [54], [55] (Table 1). Importantly, WTAP is necessary for the activity of m^6^A writer complex [52], [54], [55]. Knockdown of *WTAP* significantly reduces endogenous m^6^A levels in human cell lines [52], [54], [55]. The RNA-binding ability of METTL3 is substantially reduced in the absence of WTAP, indicating that WTAP is likely to be responsible for recruiting other m^6^A writers to target RNAs [54]. Furthermore, WTAP plays a crucial role in methyltransferase localization. *WTAP* depletion decreases the accumulation of both METTL3 and METTL14 in nuclear speckles enriched with pre-mRNA processing factors [54]. In addition, WTAP affects the fate of RNA by regulating alternative splicing associated with METTL3 and promotes transcript decay in a manner dependent on the WTAP-induced m^6^A methylation [55]. These results provide convincing evidence that WTAP functions as a regulatory component in the m^6^A writer complex and is important for generating the distinct landscape of mRNA methylation.

KIAA1429 and RBM15 have been reported to interact with WTAP as part of a novel complex regulating pre-mRNA splicing [70]. Subsequent studies indicate that these proteins also regulate m^6^A formation as a part of the methyltransferase complex [42], [55]. KIAA1429 is required for the full methylation program in mammals, and *KIAA1429* silencing causes a substantial reduction in m^6^A levels [55]. KIAA1429 mediates the methylation events near 3′UTR and stop codon of mRNAs [71]. Virilizer (the homolog of KIAA1429 in *Drosophila*) associates with both inducer of meiosis 4 (IME4, a homolog of METTL3 in *Drosophila*) and female-lethal (2)d (Fl(2)d, a homolog of WTAP in *Drosophila*) to form the m^6^A methylation complex that controls sex determination [25], [26].

RBM15 and its paralog, RBM15B, are additional subunits in the methyltransferase complex as reported in recent studies [42]. In human embryonic kidney 293T (HEK293T) cells, knockdown of *RBM15* and *RBM15B* decreases m^6^A levels and impairs X-inactive specific transcript (*XIST*)-mediated gene silencing, illustrating the importance of these proteins in the female mammalian development. In addition, coimmunoprecipitation analyses reveal that the interactions of RBM15 and RBM15B with METTL3 depend on WTAP in mammalian cells [42]. Studies in *Drosophila* provide further support for these findings by showing that Spenito (a homolog of RBM15 in *Drosophila*) is necessary for m^6^A formation [13], [25]. During development, the *Spenito* mRNA expression level correlates with the m^6^A abundance, and *Spenito* knockdown leads to an acute reduction in m^6^A levels. Thus, KIAA1429 and RBM15/RBM15B are *bona fide* components of the methyltransferase complex [12], [13], [25], [26], [27]. However, due to the structural limitation, the molecular mechanism of how WTAP, KIAA1429, and RBM15/RBM15B precisely regulate the m^6^A pathway remains unknown.

Moreover, other proteins like zinc finger CCCH domain-containing protein 13 (ZC3H13) and E3 ubiquitin-protein ligase Hakai (HAKAI) have been reported to interact with the methyltransferase complex components and affect the methylation pathway [69], [71], [72], [73], [74], [75], [76]. For instance, METTL3 regulates miRNA maturation by interacting with DiGeorge syndrome chromosomal region 8 (DGCR8), which binds to Drosha, an RNase III, and forms the microprocessor complex that cleaves the pri-miRNA [43]. Another example is zinc finger protein 217 (ZFP217), which is involved in somatic cell reprogramming [73]. METTL3 interacts with ZFP217 and protects pluripotent RNAs from rapid degradation by restraining the m^6^A RNA modification, thereby enabling embryonic stem cells to remain pluripotent and undergo reprogramming [74].

## ALKBH5 and FTO are termed m^6^A erasers

AlkB homolog 5 (ALKBH5) [28] and the fat mass and obesity-associated protein (FTO) [56] demethylate m^6^A in RNA, thus acting as erasers. Both enzymes belong to the 2-oxoglutarate (2OG)-dependent oxygenase family [77], [78], which comprises more than 60 predicted members in the human genome, including ten-eleven translocation family (TETs) [79], [80], [81], [82], [83]. Enzymes in this family are involved in DNA repair, RNA hydroxylation, and 5-methylcytosine oxidation [79], [80], [82], [83], [84], [85]. The consensus catalytic mechanism of this family is substrate hydroxylation [81]. Interestingly, although ALKBH5 and FTO exhibit similar substrate preferences when demethylating m^6^A on single-stranded RNA (ssRNA) [28], [56], their reaction pathways are different. ALKBH5 directly converts m^6^A to adenosine, whereas FTO successively demethylates m^6^A through two intermediates, *N*^6^-hydroxymethyladenosine (hm^6^A) and *N*^6^-formyladenosine (fm^6^A) [86]. Additionally, FTO also demethylates 3-methylthymine (3meT) in single-stranded DNA (ssDNA) [87], 3-methyluracil (3-meU) [87], and *N*^6^,2′-*O*-dimethyladenosine (m^6^A_m_) in ssRNA [88].

ALKBH5 localizes with nuclear speckles and functions in regulating splicing and export of nuclear RNA, gene expression, and testis development [28], [35]. *ALKBH5* is highly expressed in spermatogenic cells and some tumor cells [35], [89]. Deficiency of *ALKBH5* leads to a global increase in m^6^A in mRNAs [28]. FTO, which is found in both nucleus and cytoplasm [90], has been reported to regulate mRNA splicing, gene expression, and cell differentiation [34], [56], [91], [92]. *FTO* is widely expressed in adult and fetal tissues in brain and cancer cells [33], [91], [93]. These findings suggest that ALKBH5 and FTO play critical roles in posttranscriptional control of mammalian cell development [12], [13], [33].

**Figure 2:**
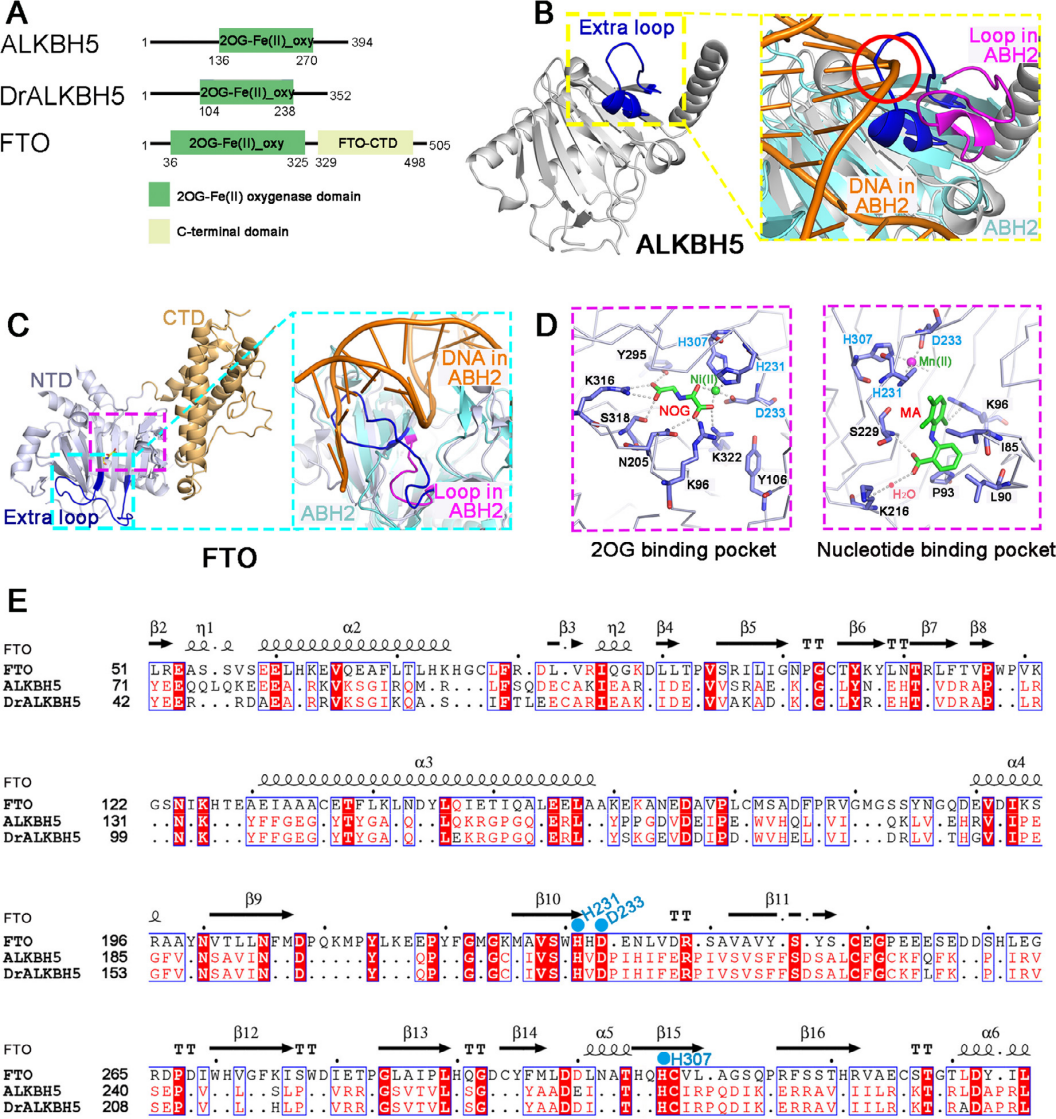
**Structural insights into ALKBH5 and FTO as m^6^A erasers** **A.** Domain architectures of m^6^A eraser proteins for ALKBH5 (GenBank accession: Q6P6C2.2), DrALKBH5 (GenBank accession: NP_001070855.1), and FTO (GenBank accession: Q9C0B1.3). **B.** Overall structure of ALKBH5 (PDB ID: 4NJ4) and a detailed view of the extra loop against dsDNA. The overall and detailed structure of ALKBH5 is colored in gray. The detailed view of the extra loop (blue) is highlighted in the yellow dashed box, and the partial structure of ABH2 is colored in cyan. The overall structure of ALKBH5 is well superimposed onto that of ABH2, except the extra loop, which clashes with the substrate DNA (highlighted in the red circle) in ALKBH5 when compared to the equivalent loop (purple) in ABH2 (PDB ID: 3RZG). **C.** Overall structure of FTO (PDB ID: 4QKN) and a detailed view of the extra loop against dsDNA in FTO. The NTD of FTO is colored in light gray, and the CTD is colored in light brown. The detailed view of the extra loop (blue) is highlighted in the cyan dashed box. The FTO-NTD (light gray) is well superimposed on ABH2 (cyan) (PDB ID: 3RZG). The extra loop (blue) in FTO-NTD forms a barrier for its selection against dsDNA/RNA. **D.** Interaction networks between the FTO protein and inhibitors in its 2OG-binding pocket and nucleotide-binding pocket. Residues involved in the interactions are shown as sticks, and residues in the conserved motif (HXD…H) are colored in cyan. The structure of the 2OG-binding pocket bound to a 2OG analog, NOG, is shown in the dashed box on the left (PDB ID: 4QKN), and the structure of nucleotide-binding pocket bound to a compound, MA, is shown in the dashed box on the right (PDB ID: 4QKN). **E.** Sequence alignments of human FTO, human ALKBH5, and DrALKBH5 proteins. The secondary structure of FTO is shown on the top with the detailed amino acid sequences shown below. Residues in the conserved motif (HXD…H) of FTO that coordinate the active site metal are highlighted in cyan. ABH2, AlkB homolog 2; CTD, C-terminal domain; DrALKBH5, *Danio rerio* ALKBH5; MA, meclofenamic acid; NOG, *N*-oxalylglycine; NTD: N-terminal domain; 2OG, 2-oxoglutarate; oxy, oxygenase.

In early 2014, four groups independently reported the structures of human or zebrafish ALKBH5 with or without ligands [78], [86], [94], [95] (Table 2 and Figure 2A and B). The overall structure of ALKBH5 displays a typical jellyroll fold [85]. The catalytic metal is coordinated by the conserved HXD…H motif. The motif consists of AA residues H204, D206, and H266 [78], [86], [94], [95], which are essential for demethylation activity [28], [36]. The structure of ALKBH5 are well superimposed onto the structure of AlkB homolog 2 (ABH2), which functions as the primary protective enzyme in mammalian cells that efficiently repairs endogenously alkylated lesions in double-stranded DNA (dsDNA) [96]. However, a large extra loop (AA residues 229–242) in ALKBH5 shows a potential steric clash with the duplex DNA (Figure 2B). Thus, ALBKH5 strongly prefers ssRNA as a substrate, a prediction that is consistent with biochemical observations. Additionally, the extra loop is stabilized by C230 and C267 through the formation of a disulfide bond, which is essential for ssRNA recognition [94], [95]. Disruption of the disulfide bond results in an apparent affinity for dsDNA [95].

The structure of FTO was first determined by Chai’s group [77] (Table 2 and Figure 2C). FTO contains two well-defined domains: the N-terminal domain (AA residues 32–326; referred to as NTD) and the C-terminal domain (residues 327–498; referred to as CTD) [77]. As the catalytic domain, NTD exhibits a classic jellyroll fold. Similar to ALKBH5, three residues, H231, D233, and H307, constitute the HXD…H motif to coordinate the catalytic metal atom [77], [80], [97], [98] (Figure 2D and E). The unique CTD primarily functions in stabilizing the architecture of the NTD. Coincidently, FTO also possesses an extra loop (AA residues 213–224), which strongly excludes the DNA duplex from the catalytic center and is important for the selectivity of FTO against dsDNA/RNA [77]. Notably, although both ALKBH5 and FTO have an extra loop required for ssRNA binding, the loop of ALKBH5 extends from the opposite direction compared to FTO.

Due to its vital role in human obesity, FTO is a potential drug target [77], [99]. Several groups have developed sets of FTO inhibitors targeting the 2OG-binding pocket or the nucleotide-binding pocket [80], [97], [98] (Figure 2D). Schofield and colleagues have screened various 2OG derivatives and related compounds [80], and identified a set of cyclic and acyclic 2OG derivatives as FTO inhibitors. They reported six structures for FTO in complex with *N*-oxalylglycine (NOG; PDB ID: 4IDZ), pyridine-2,4-dicarboxylate (PD2; PDB ID: 4IE0), 8-hydroxyquinolines (8XQ; PDB ID: 4IE4), 2-(3-hydroxypicolinamido) acetic acid (MD6; PDB ID: 4IE5), (1-chloro-4-hydroxyisoquinoline3-carbonyl) glycine (IOX3; PDB: 4IE6), and rhein (PDB ID: 4IE7) (Table 2). NOG, PD2, and 8XQ are completely trapped in the 2OG-binding pocket, whereas MD6 and IOX3 are not only located in the 2OG-binding pocket of FTO through interactions with their carboxylate side chain but also insert their various bulky chains into the adjacent nucleotide-binding pocket, thus blocking the entrance of its nucleotide substrate [80]. The last compound, rhein, binds to the nucleotide-binding pocket rather than the predicted 2OG-binding site [80], [100].

In other examples, Yang’s group has characterized the interaction of meclofenamic acid (MA), a non-steroidal and anti-inflammatory drug, with the nucleotide-binding pocket of FTO [97] (Figure 2D). Based on their results from fluorescence polarization and fluorescence-based thermal shift assays, MA is likely to compete with m^6^A-containing nucleic acids for FTO binding [97]. It is of note that rhein and MA do not specifically inhibit FTO over other enzymes. For example, rhein also binds to 2OG-binding site of AlkB, a dioxygenase from *Escherichia coli*, and inhibits its activity [101]. As an anti-inflammatory drug, MA binds to the catalytic center of aldo–keto reductase 1C3 (AKR1C3) protein, which catalyzes the reduction of carbonyl groups on both steroids and prostaglandins in humans [102]. In collaboration with Zhou’s group, they have further developed several fluorescein derivatives as bifunctional molecules for the simultaneous inhibition and photoaffinity labeling of the FTO protein [98] (Table 2). Among the 12 compounds (named FL1–12) initially designed and synthesized, extensive biochemical screening has shown that FL1–8 completely inhibits the demethylation activity of FTO *in vitro*. The authors have further determined high-resolution structures of the FTO/FL1 and FTO/FL4 complexes to gain insights into the interaction modes between FTO and the derivatives, and have revealed that the fluorescein derivatives are stabilized in the nucleotide-binding pocket [98]. Taken together, these findings provide structural insights that would further our understanding of the substrate recognition specificity of ALKBH5 and FTO, and offer a framework for the design of selective inhibitors.

## YTH family proteins serve as m^6^A readers

**Figure 3:**
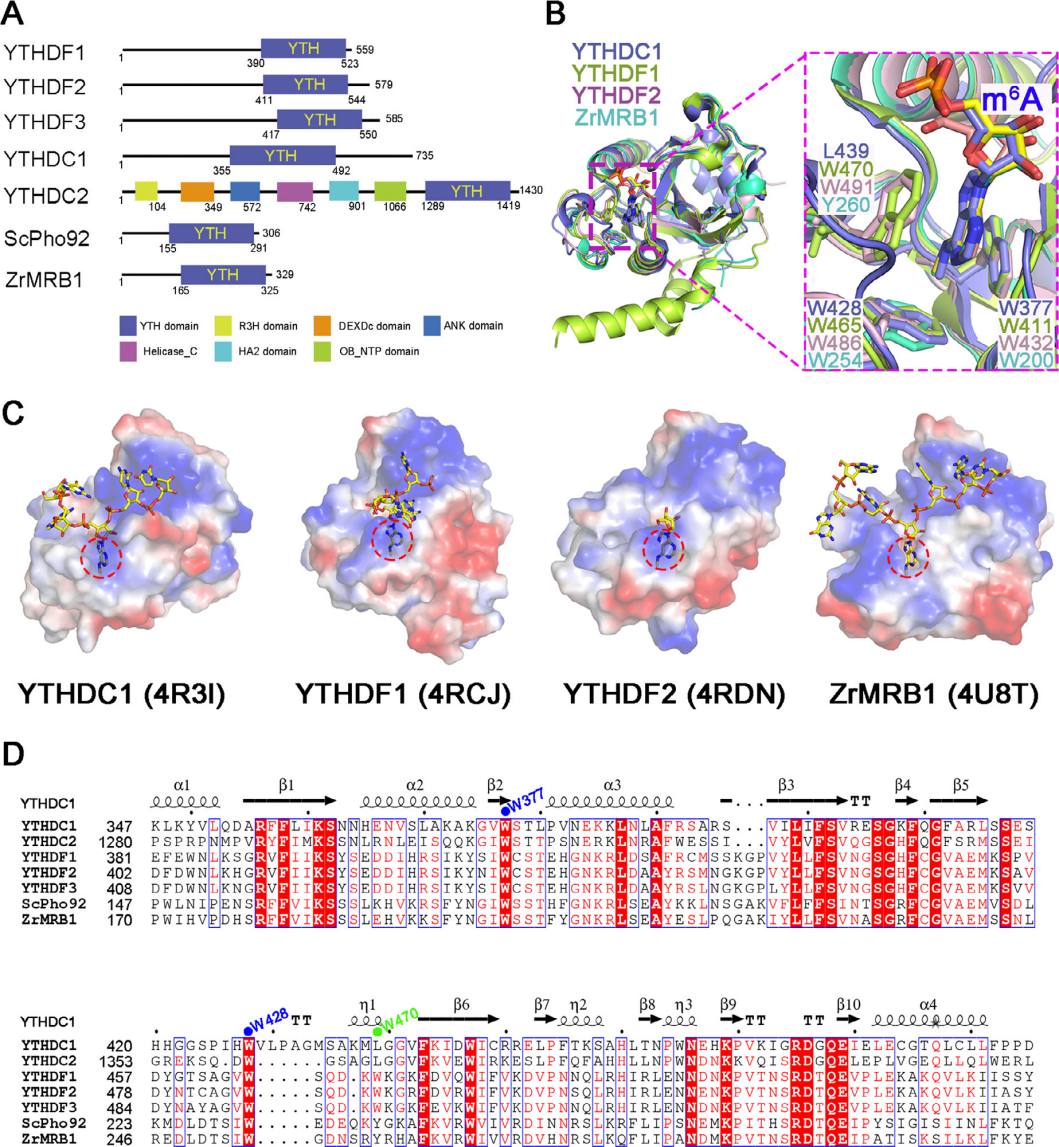
**Structural insights into the YTH family proteins as m^6^A readers** **A.** Domain architectures of the YTH family proteins. YTHDC1 (GenBank accession: NP_001317627.1); YTHDC2 (GenBank accession: NP_073739.3); YTHDF1 (GenBank accession: Q9BYJ9.1); YTHDF2 (GenBank accession: Q9Y5A9.2); YTHDF3 (GenBank accession: Q7Z739.1); ScPho92 (GenBank accession: Q06390.1); and ZrMRB1 (GenBank accession: XP_002498076.1). **B.** Superposition of the crystal structures of YTHDC1 (PDB ID: 4R3I), YTHDF1 (PDB ID: 4RCJ), YTHDF2 (PDB ID: 4RDN), and ZrMRB1 (PDB ID: 4U8T). The overall structures of these proteins are displayed in cartoon mode, and the conserved tryptophan (W) residues are shown as sticks. The conserved aromatic cage is highlighted in the purple dashed box. **C.** The electrostatic surface of the YTH domain in complex with the m^6^A-containing RNA. Shown from left to right are YTHDC1 (PDB ID: 4R3I), YTHDF1 (PDB ID: 4RCJ), YTHDF2 (PDB ID: 4RDN), and ZrMRB1 (PDB ID: 4U8T), respectively. The m^6^A-containing RNAs are shown as sticks. The aromatic cage accommodating the methylated adenosine is highlighted with a red dashed circle. **D.** Sequence alignments of human YTH family proteins and yeast YTH proteins. The secondary structure of human YTHDC1 is shown on the top with the detailed amino acid sequences shown below. The conserved tryptophan residues that form the aromatic cage in YTHDC1 and YTHDF1 are indicated by blue and green dots, respectively. ANK domain, ankyrin repeat domain; DEXDc, DEAD-like helicases superfamily; Helicase_C domain: helicase conserved C-terminal domain; HA2 domain, helicase associated domain; OB_NTP domain, oligonucleotide/oligosaccharide-binding (OB)-fold domain; R3H domain, ATP-dependent DNA or RNA helicase domain containing conserved arginine and histidine residues; ScPho92, *Saccharomyces cerevisiae* Pho92 protein; YTHDC, YTH domain-containing protein; YTHDF, YTH domain-containing family protein; ZrMRB1, *Zygosaccharomyces rouxii* methylated RNA-binding protein 1.

YTH family proteins are well-documented m^6^A readers that can specifically recognize m^6^A-containing RNAs through the conserved YTH domain [3], [13], [49]. In mammals, there are five members in the YTH family, *i.e.*, YTHDC1, YTHDC2, YTHDF1, YTHDF2, and YTHDF3 [60], [103] (Figure 3A), which all prefer to bind to m^6^A-containing RNAs. Rechavi and colleagues first identified YTHDF2 and YTHDF3 using m^6^A RNA pulldown assay [3]. Later, He and colleagues discovered that YTHDF1 is another m^6^A-selective binding protein [104]. Then, using a gel shift assay, Min and colleagues revealed that YTHDC1 preferentially binds to methylated RNA compared with the unmethylated control [105]. YTHDC2 preferentially binds to m^6^A-containing RNA, as shown by recent studies from two independent groups using an *in vitro* pulldown assay and fluorescence anisotropy, respectively [60], [103]. All five members directly target m^6^A-containing mRNAs and affect their fates, such as splicing, stability, and translational efficiency. YTHDC1, which is localized in the nucleus, can regulate mRNA splicing by recruiting pre-mRNA splicing factors [106], [107], get involved into *XIST*-mediated gene silencing [42], and affect export efficiency of methylated mRNAs from the nucleus [108]. Conversely, the other four members exert their functions in the cytoplasm. Among them, YTHDF1, YTHDF3, and YTHDC2, together or alone, selectively enhance the translation efficiency of m^6^A-containing mRNAs [3], [60], [103], [104], [109], [110], [111], whereas YTHDF2 interacts with the CCR4-NOT deadenylase complex to decrease mRNA stability [19], [104], [112].

The structure of YTH family proteins from different species has been reported by several groups [105], [113], [114], [115], [116], [117] (Table 2, Figure 3B and C). The overall structure of the YTH domain exhibits a sphere-like fold, with a central core of 4–6 parallel β-sheets surrounded by 4–8 α-helixes [105]. Based on their structural features, YTH domains belong to the pseudouridine synthase and archaeosine transglycosylase (PUA)-like superfamily [113], [118], [119], which primarily adopts an open α/β fold. Notably, YTH domains contain several conserved tryptophan residues [W377, W428, and L439 in YTHDC1; W411, W465, and W470 in YTHDF1; W432, W486, and W491 in YTHDF2; as well as W200, W254, and Y260 in methylated RNA-binding protein 1 (ZrMRB1, an YTH protein homolog in *Zygosaccharomyces rouxii*)] that form an aromatic cage to accommodate the m^6^A [105], [113], [114], [116], [117] (Figure 3B–D). The methylated adenosine is well recognized by π-π interactions between the adenine base and the aromatic walls and cation-π interactions between the *N*^6^-methyl moiety and the aromatic cage. Moreover, a set of hydrogen bonds also contributes to this recognition mode [105], [113], [116]. Mutations of aromatic cage residues dramatically decrease the binding affinity of the YTH domain to the methylated RNA probe, but these mutations rarely affect the binding affinity to unmethylated RNA [115]. The structural features of the conserved aromatic cage explain the specificity of m^6^A reading.

YTH domains also interact with the nucleotides preceding and following m^6^A [105], [113], [114], [117]. For example, YTHDC1 favors a guanosine over adenosine at the −1 position relative to the m^6^A because the main chain NH group of V382 forms a hydrogen bond with the carbonyl oxygen of guanine (6-oxo). Replacement of the guanosine at the −1 position with any other base would disrupt the hydrogen bond. Likewise, YTHDC1 slightly prefers guanosine and cytidine at the −2 and +1 positions, respectively, due to the formation of specific hydrogen bonds [105]. These structural observations unveil the sequence preference of YTH readers for the RRACH motif, which is also a substrate sequence for the writer complex.

Recently, two groups reported the structure of the RNA-bound YTH domain of meiotic mRNA interception protein 1 (Mmi1) [120], [121] from *Schizosaccharomyces pombe* [122], [123]. Although the YTH domain of Mmi1 superimposes well onto that of YTHDC1, the RNA-binding surfaces of the two proteins are distinct. YTHDC1 recognizes the m^6^A RNA probe via an aromatic cage, whereas Mmi1 binds to the target RNA via a long groove, which opposes its aromatic cage. Moreover, the region of Mmi1 neighboring the aromatic cage is enriched with a set of negatively-charged residues (D358, D360, D423, E441, and D453), generating a severely-repulsive surface to potentially abrogate the m^6^A RNA-binding activity [122]. Interestingly, this hypothesis is corroborated by biochemical studies showing that the aromatic cage of Mmi1 does not bind to m^6^A-containing RNAs [122], [123]. These investigations about Mmi1 have expanded our knowledge of the YTH domains.

## Conclusions and perspectives

Although tremendous progress has been achieved in exploring the molecular mechanisms underlying reversible modification and m^6^A recognition both in *vivo* and in *vitro* in recent years, some questions and debates remain [56], [88], [124], [125].

First, how the consensus mRNA sequence is specifically recognized by the writer complex is poorly understood. Bioinformatics analyses reveal that two CCCH-type zinc finger domains (ZFDs) are present preceding the MTD in the N-terminus of METTL3 [62], and biochemistry assay proved that the ZFDs are necessary for the methylation activity [31], [64]. Recently we have determined the solution structure of ZFD [126]. The ZFD serves as the target recognition domain and specifically targets to GGACU-containing RNAs [126]. Besides, the secondary structure of mRNA might affect the writer’s catalyzing activity [52] *in vivo*. More importantly, further investigations are required to examine whether these regulators could spatiotemporally regulate the m^6^A modification and how they function in determining the localization of m^6^A during various cellular processes. Structural examinations of the writer complex in the presence or absence of RNA may provide insights to unambiguously clarify the underlying molecular mechanisms. In addition, METTL16 is another m^6^A-forming enzyme that can methylate pre-mRNAs, lncRNAs, and other ncRNAs [127], [128]. The molecular mechanisms of METTL16 also deserve to be further explored.

Second, although the structures of erasers like FTO and ALKBH5 have been determined, the mechanism by which these enzymes specifically recognize m^6^A-containing RNAs remains unknown, due to the lack of structural information of protein–RNA complexes. The complex structure of FTO containing 1-mer 3-meT nucleotide may provide some insights into the m^6^A recognition. Notably, previous studies have shown that neither ALKBH5 nor FTO prefers to bind methylated consensus-sequence-containing RNA *in vitro* [129]. Recently Huang’s group has identified one lncRNA, FOXM1-AS, antisense to *FOXM1*, that can facilitate the interaction between ALKBH5 and nascent *FOXM1* transcripts [89], suggesting that ALKBH5 might function in a sequence-specific context. Furthermore, by combining CLIP-seq and RNA-seq methods, Vanacova’s group shows that FTO regulates several mRNA metabolic events such as alternative splicing and 3′-end processing [34]. Interestingly, the CLIP-seq data reveal that FTO is de-enriched in m^6^A target sequences [34]. This raises the question whether FTO binds to RRACH motif-containing RNA *in vivo*. Additionally, the m^6^A_m_ modification that is typically present at the first nucleotide in mRNAs is also a reversible modification that affects the fate of the mRNAs [19], [130], [131]. Interestingly, FTO has recently been shown to erase m^6^A_m_ in an m^7^G cap-dependent manner [88]. This finding expands our knowledge of the biological functions of the m^6^A eraser FTO. Thus, the mechanism by which FTO erases m^6^A_m_ is also worth exploring.

In addition to the YTH family proteins, several RNA-binding proteins, such as the fragile X-linked mental retardation syndrome protein 1 (FMR1) [132], eukaryotic initiation factors 3 (eIF3) [133], insulin-like growth factor 2 mRNA-binding proteins (IGF2BPs) [134], and heterogeneous nuclear ribonucleoproteins A2/B1 (hnRNPA2/B1) [135], also bind to m^6^A-containing RNAs. For example, the FMR1 protein has recently been described as an m^6^A reader that regulates mRNA homeostasis in a sequence context-dependent manner. FMR1 can bind to some mRNAs as a negative regulator to affect their translation. Using the immunoprecipitation assay *in vivo*, Edupuganti et al. indicate that FMR1 is colocalized with m^6^A on mRNA and favorably interact with m^6^A-methylated mRNAs [132]. Given there are no YTH domains in FMR1, whether FMR1 interacts with m^6^A-methylated mRNAs in a direct or indirect way remains unknown; therefore, the precise mechanism by which FMR1 recognizes methylated RNA needs further investigation. Other potential m^6^A-containing RNA-binding proteins also have distinct sequence organizations compared to YTH proteins. Ma and colleagues find that hnRNPA2/B1, which appears to be an m^6^A reader as reported in a previous study [135], does not prefer to recognize m^6^A-containing RNAs compared to the unmethylated ones [136]. They speculate that hnRNPA2/B1 may regulate the functions of m^6^A modification via an “m^6^A switch” mechanism, by which m^6^A induces RNA unfolding for the access of RBPs [136]. Whether these proteins function as *bona fide* m^6^A readers in the m^6^A signaling pathway and how they do if yes are among other questions to be answered.

Last, but not least, we only have limited information on the roles of m^6^A methylation in other species and whether the m^6^A writers, readers, erasers are conserved evolutionarily. ALKBH10B, an m^6^A demethylase in *Arabidopsis*, plays a crucial role in development by delaying flowering and repressing vegetative growth [24]. Using genetic screening, the “MIS” complex have been identified as a core RNA methyltransferase in yeast, which comprises Mum2 (orthologous to mammalian WTAP), Ime4 (orthologous to mammalian METTL3), and sporulation-specific with a leucine zipper motif protein 1 (Slz1), a third ancillary factor [20], [22]. The MIS complex delays meiosis by abrogating mRNA methylation activity in yeast [20], [21], [22]. Further studies are warranted to address the discrepancies on the ways these components get involved in m^6^A modification among various species.

Notably, although m^6^A was initially identified more than 40 years ago, it only becomes a research hotspot in epigenetics in the last six years. “*What's past is prologue*”: we would expect to see an increasing number of studies on the mechanisms underlying m^6^A modification.

## Competing interests

The authors have declared no competing interests.

## References

[1] Boccaletto P., Machnicka M.A., Purta E., Piatkowski P., Baginski B., Wirecki T.K. (2018). MODOMICS: a database of RNA modification pathways. 2017 update. Nucleic Acids Res.

[2] Li X., Xiong X., Yi C. (2016). Epitranscriptome sequencing technologies: decoding RNA modifications. Nat Methods.

[3] Dominissini D., Moshitch-Moshkovitz S., Schwartz S., Salmon-Divon M., Ungar L., Osenberg S. (2012). Topology of the human and mouse m^6^A RNA methylomes revealed by m^6^A-seq. Nature.

[4] Meyer K.D., Saletore Y., Zumbo P., Elemento O., Mason C.E., Jaffrey S.R. (2012). Comprehensive analysis of mRNA methylation reveals enrichment in 3' UTRs and near stop codons. Cell.

[5] Motorin Y., Lyko F., Helm M. (2010). 5-methylcytosine in RNA: detection, enzymatic formation and biological functions. Nucleic Acids Res.

[6] Squires J.E., Patel H.R., Nousch M., Sibbritt T., Humphreys D.T., Parker B.J. (2012). Widespread occurrence of 5-methylcytosine in human coding and non-coding RNA. Nucleic Acids Res.

[7] Li X., Xiong X., Zhang M., Wang K., Chen Y., Zhou J. (2017). Base-resolution mapping reveals distinct m(1)A methylome in nuclear- and mitochondrial-encoded transcripts. Mol Cell.

[8] Safra M., Sas-Chen A., Nir R., Winkler R., Nachshon A., Bar-Yaacov D. (2017). The m^1^A landscape on cytosolic and mitochondrial mRNA at single-base resolution. Nature.

[9] Schwartz S., Bernstein D.A., Mumbach M.R., Jovanovic M., Herbst R.H., Leon-Ricardo B.X. (2014). Transcriptome-wide mapping reveals widespread dynamic-regulated pseudouridylation of ncRNA and mRNA. Cell.

[10] Carlile T.M., Rojas-Duran M.F., Zinshteyn B., Shin H., Bartoli K.M., Gilbert W.V. (2014). Pseudouridine profiling reveals regulated mRNA pseudouridylation in yeast and human cells. Nature.

[11] Levanon E.Y., Eisenberg E., Yelin R., Nemzer S., Hallegger M., Shemesh R. (2004). Systematic identification of abundant A-to-I editing sites in the human transcriptome. Nat Biotechnol.

[12] Roundtree I.A., Evans M.E., Pan T., He C. (2017). Dynamic RNA modifications in gene expression regulation. Cell.

[13] Meyer K.D., Jaffrey S.R. (2017). Rethinking m(6)A readers, writers, and erasers. Annu Rev Cell Dev Biol.

[14] Lewis C.J., Pan T., Kalsotra A. (2017). RNA modifications and structures cooperate to guide RNA-protein interactions. Nat Rev Mol Cell Biol.

[15] Roundtree I.A., He C. (2016). RNA epigenetics–chemical messages for posttranscriptional gene regulation. Curr Opin Chem Biol.

[16] Desrosiers R., Friderici K., Rottman F. (1974). Identification of methylated nucleosides in messenger RNA from Novikoff hepatoma cells. Proc Natl Acad Sci U S A.

[17] Courtney D.G., Kennedy E.M., Dumm R.E., Bogerd H.P., Tsai K., Heaton N.S. (2017). Epitranscriptomic enhancement of influenza a virus gene expression and replication. Cell Host Microbe.

[18] Martinez-Perez M., Aparicio F., Lopez-Gresa M.P., Belles J.M., Sanchez-Navarro J.A., Pallas V. (2017). *Arabidopsis* m(6)A demethylase activity modulates viral infection of a plant virus and the m(6)A abundance in its genomic RNAs. Proc Natl Acad Sci U S A.

[19] Tan B., Liu H., Zhang S., da Silva S.R., Zhang L., Meng J. (2018). Viral and cellular *N*(6)-methyladenosine and *N*(6),2'-*O*-dimethyladenosine epitranscriptomes in the KSHV life cycle. Nat Microbiol.

[20] Agarwala S.D., Blitzblau H.G., Hochwagen A., Fink G.R. (2012). RNA methylation by the MIS complex regulates a cell fate decision in yeast. PLoS Genet.

[21] Schwartz S., Agarwala S.D., Mumbach M.R., Jovanovic M., Mertins P., Shishkin A. (2013). High-resolution mapping reveals a conserved, widespread, dynamic mRNA methylation program in yeast meiosis. Cell.

[22] Yadav P.K., Rajasekharan R. (2017). The m(6)A methyltransferase Ime4 epitranscriptionally regulates triacylglycerol metabolism and vacuolar morphology in haploid yeast cells. J Biol Chem.

[23] Zhong S., Li H., Bodi Z., Button J., Vespa L., Herzog M. (2008). MTA is an *Arabidopsis* messenger RNA adenosine methylase and interacts with a homolog of a sex-specific splicing factor. Plant Cell.

[24] Duan H.C., Wei L.H., Zhang C., Wang Y., Chen L., Lu Z. (2017). ALKBH10B is an RNA *N*^6^-methyladenosine demethylase affecting *Arabidopsis* floral transition. Plant Cell.

[25] Lence T., Akhtar J., Bayer M., Schmid K., Spindler L., Ho C.H. (2016). m(6)A modulates neuronal functions and sex determination in *Drosophila*. Nature.

[26] Haussmann I.U., Bodi Z., Sanchez-Moran E., Mongan N.P., Archer N., Fray R.G. (2016). m(6)A potentiates Sxl alternative pre-mRNA splicing for robust *Drosophila* sex determination. Nature.

[27] Kan L., Grozhik A.V., Vedanayagam J., Patil D.P., Pang N., Lim K.S. (2017). The m(6)A pathway facilitates sex determination in *Drosophila*. Nat Commun.

[28] Zheng G., Dahl J.A., Niu Y., Fedorcsak P., Huang C.M., Li C.J. (2013). ALKBH5 is a mammalian RNA demethylase that impacts RNA metabolism and mouse fertility. Mol Cell.

[29] Yoon K.J., Ringeling F.R., Vissers C., Jacob F., Pokrass M., Jimenez-Cyrus D. (2017). Temporal control of mammalian cortical neurogenesis by m(6)A methylation. Cell.

[30] Jain D., Puno M.R., Meydan C., Lailler N., Mason C.E., Lima C.D. (2018). *ketu* mutant mice uncover an essential meiotic function for the ancient RNA helicase YTHDC2. Elife.

[31] Wang X., Huang J., Zou T., Yin P. (2017). Human m(6)A writers: two subunits, 2 roles. RNA Biol.

[32] Wang S., Sun C., Li J., Zhang E., Ma Z., Xu W. (2017). Roles of RNA methylation by means of *N*(6)-methyladenosine (m(6)A) in human cancers. Cancer Lett.

[33] Deng X., Su R., Feng X., Wei M., Chen J. (2017). Role of *N*(6)-methyladenosine modification in cancer. Curr Opin Genet Dev.

[34] Bartosovic M., Molares H.C., Gregorova P., Hrossova D., Kudla G., Vanacova S. (2017). *N*^6^-methyladenosine demethylase FTO targets pre-mRNAs and regulates alternative splicing and 3'-end processing. Nucleic Acids Res.

[35] Tang C., Klukovich R., Peng H., Wang Z., Yu T., Zhang Y. (2018). ALKBH5-dependent m^6^A demethylation controls splicing and stability of long 3'-UTR mRNAs in male germ cells. Proc Natl Acad Sci U S A.

[36] Zheng Q., Hou J., Zhou Y., Li Z., Cao X. (2017). The RNA helicase DDX46 inhibits innate immunity by entrapping m(6)A-demethylated antiviral transcripts in the nucleus. Nat Immunol.

[37] Vu L.P., Pickering B.F., Cheng Y., Zaccara S., Nguyen D., Minuesa G. (2017). The *N*(6)-methyladenosine (m(6)A)-forming enzyme METTL3 controls myeloid differentiation of normal hematopoietic and leukemia cells. Nat Med.

[38] Bertero A., Brown S., Madrigal P., Osnato A., Ortmann D., Yiangou L. (2018). The SMAD2/3 interactome reveals that TGFbeta controls m(6)A mRNA methylation in pluripotency. Nature.

[39] Barbieri I., Tzelepis K., Pandolfini L., Shi J., Millan-Zambrano G., Robson S.C. (2017). Promoter-bound METTL3 maintains myeloid leukaemia by m(6)A-dependent translation control. Nature.

[40] Zhou J., Wan J., Shu X.E., Mao Y., Liu X.M., Yuan X. (2018). *N*(6)-methyladenosine guides mRNA alternative translation during integrated stress response. Mol Cell.

[41] Coots R.A., Liu X.M., Mao Y., Dong L., Zhou J., Wan J. (2017). m(6)A facilitates eIF4F-independent mRNA translation. Mol Cell.

[42] Patil D.P., Chen C.K., Pickering B.F., Chow A., Jackson C., Guttman M. (2016). m(6)A RNA methylation promotes *XIST*-mediated transcriptional repression. Nature.

[43] Alarcon C.R., Lee H., Goodarzi H., Halberg N., Tavazoie S.F. (2015). *N*^6^-methyladenosine marks primary microRNAs for processing. Nature.

[44] Niu Y., Zhao X., Wu Y.S., Li M.M., Wang X.J., Yang Y.G. (2013). *N*^6^-methyl-adenosine (m^6^A) in RNA: an old modification with a novel epigenetic function. Genomics Proteomics Bioinformatics.

[45] Batista P.J. (2017). The RNA modification *N*(6)-methyladenosine and its implications in human disease. Genomics Proteomics Bioinformatics.

[46] Visvanathan A., Somasundaram K. (2018). mRNA traffic control reviewed: *N*^6^-methyladenosine (m(6) A) takes the driver's seat. Bioessays.

[47] Wu B., Li L., Huang Y., Ma J., Min J. (2017). Readers, writers and erasers of *N*(6)-methylated adenosine modification. Curr Opin Struct Biol.

[48] Aguilo F., Walsh M.J. (2017). The *N*(6)-methyladenosine RNA modification in pluripotency and reprogramming. Curr Opin Genet Dev.

[49] Patil D.P., Pickering B.F., Jaffrey S.R. (2018). Reading m(6)A in the transcriptome: m(6)A-binding proteins. Trends Cell Biol.

[50] Schibler U., Kelley D.E., Perry R.P. (1977). Comparison of methylated sequences in messenger RNA and heterogeneous nuclear RNA from mouse L cells. J Mol Biol.

[51] Wei C.M., Moss B. (1977). Nucleotide sequences at the *N*^6^-methyladenosine sites of HeLa cell messenger ribonucleic acid. Biochemistry.

[52] Liu J., Yue Y., Han D., Wang X., Fu Y., Zhang L. (2014). A METTL3-METTL14 complex mediates mammalian nuclear RNA *N*^6^-adenosine methylation. Nat Chem Biol.

[53] Wang Y., Li Y., Toth J.I., Petroski M.D., Zhang Z., Zhao J.C. (2014). *N*^6^-methyladenosine modification destabilizes developmental regulators in embryonic stem cells. Nat Cell Biol.

[54] Ping X.L., Sun B.F., Wang L., Xiao W., Yang X., Wang W.J. (2014). Mammalian WTAP is a regulatory subunit of the RNA *N*^6^-methyladenosine methyltransferase. Cell Res.

[55] Schwartz S., Mumbach M.R., Jovanovic M., Wang T., Maciag K., Bushkin G.G. (2014). Perturbation of m^6^A writers reveals two distinct classes of mRNA methylation at internal and 5' sites. Cell Rep.

[56] Jia G., Fu Y., Zhao X., Dai Q., Zheng G., Yang Y. (2011). *N*^6^-methyladenosine in nuclear RNA is a major substrate of the obesity-associated FTO. Nat Chem Biol.

[57] Bokar J.A., Rath-Shambaugh M.E., Ludwiczak R., Narayan P., Rottman F. (1994). Characterization and partial purification of mRNA *N*^6^-adenosine methyltransferase from HeLa cell nuclei. internal mRNA methylation requires a multisubunit complex. J Biol Chem.

[58] Bokar J.A., Shambaugh M.E., Polayes D., Matera A.G., Rottman F.M. (1997). Purification and cDNA cloning of the AdoMet-binding subunit of the human mRNA (*N*^6^-adenosine)-methyltransferase. RNA.

[59] Gray K.A., Yates B., Seal R.L., Wright M.W., Bruford E.A. (2015). Genenames.org: the HGNC resources in 2015. Nucleic Acids Res.

[60] Hsu P.J., Zhu Y., Ma H., Guo Y., Shi X., Liu Y. (2017). Ythdc2 is an *N*(6)-methyladenosine binding protein that regulates mammalian spermatogenesis. Cell Res.

[61] Bujnicki J.M., Feder M., Radlinska M., Blumenthal R.M. (2002). Structure prediction and phylogenetic analysis of a functionally diverse family of proteins homologous to the MT-A70 subunit of the human mRNA:m(6)A methyltransferase. J Mol Evol.

[62] Iyer L.M., Zhang D., Aravind L. (2016). Adenine methylation in eukaryotes: apprehending the complex evolutionary history and functional potential of an epigenetic modification. Bioessays.

[63] Wang X., Feng J., Xue Y., Guan Z., Zhang D., Liu Z. (2016). Structural basis of *N*(6)-adenosine methylation by the METTL3-METTL14 complex. Nature.

[64] Wang P., Doxtader K.A., Nam Y. (2016). Structural basis for cooperative function of Mettl3 and Mettl14 methyltransferases. Mol Cell.

[65] Sledz P., Jinek M. (2016). Structural insights into the molecular mechanism of the m(6)A writer complex. Elife.

[66] Bheemanaik S., Reddy Y.V., Rao D.N. (2006). Structure, function and mechanism of exocyclic DNA methyltransferases. Biochem J.

[67] Goedecke K., Pignot M., Goody R.S., Scheidig A.J., Weinhold E. (2001). Structure of the *N*^6^-adenine DNA methyltransferase M.TaqI in complex with DNA and a cofactor analog. Nat Struct Biol.

[68] Scholler E., Weichmann F., Treiber T., Ringle S., Treiber N., Flatley A. (2018). Interactions, localization and phosphorylation of the m^6^A generating METTL3-METTL14-WTAP complex. RNA.

[69] Ruzicka K., Zhang M., Campilho A., Bodi Z., Kashif M., Saleh M. (2017). Identification of factors required for m(6) A mRNA methylation in *Arabidopsis* reveals a role for the conserved E3 ubiquitin ligase HAKAI. New Phytol.

[70] Horiuchi K., Kawamura T., Iwanari H., Ohashi R., Naito M., Kodama T. (2013). Identification of Wilms' tumor 1-associating protein complex and its role in alternative splicing and the cell cycle. J Biol Chem.

[71] Yue Y., Liu J., Cui X., Cao J., Luo G., Zhang Z. (2018). VIRMA mediates preferential m(6)A mRNA methylation in 3′UTR and near stop codon and associates with alternative polyadenylation. Cell Discov.

[72] Knuckles P., Lence T., Haussmann I.U., Jacob D., Kreim N., Carl S.H. (2018). Zc3h13/Flacc is required for adenosine methylation by bridging the mRNA-binding factor Rbm15/Spenito to the m(6)A machinery component Wtap/Fl(2)d. Genes Dev.

[73] Lee D.F., Su J., Ang Y.S., Carvajal-Vergara X., Mulero-Navarro S., Pereira C.F. (2012). Regulation of embryonic and induced pluripotency by aurora kinase-p53 signaling. Cell Stem Cell.

[74] Aguilo F., Zhang F., Sancho A., Fidalgo M., Di Cecilia S., Vashisht A. (2015). Coordination of m(6)A mRNA methylation and gene transcription by ZFP217 regulates pluripotency and reprogramming. Cell Stem Cell.

[75] Wen J., Lv R., Ma H., Shen H., He C., Wang J. (2018). Zc3h13 regulates nuclear RNA m(6)A methylation and mouse embryonic stem cell self-renewal. Mol Cell.

[76] Guo J., Tang H.W., Li J., Perrimon N., Yan D. (2018). Xio is a component of the *Drosophila* sex determination pathway and RNA *N*(6)-methyladenosine methyltransferase complex. Proc Natl Acad Sci U S A.

[77] Han Z., Niu T., Chang J., Lei X., Zhao M., Wang Q. (2010). Crystal structure of the FTO protein reveals basis for its substrate specificity. Nature.

[78] Aik W., Scotti J.S., Choi H., Gong L., Demetriades M., Schofield C.J. (2014). Structure of human RNA *N*(6)-methyladenine demethylase ALKBH5 provides insights into its mechanisms of nucleic acid recognition and demethylation. Nucleic Acids Res.

[79] Rose N.R., McDonough M.A., King O.N., Kawamura A., Schofield C.J. (2011). Inhibition of 2-oxoglutarate dependent oxygenases. Chem Soc Rev.

[80] Aik W., Demetriades M., Hamdan M.K., Bagg E.A., Yeoh K.K., Lejeune C. (2013). Structural basis for inhibition of the fat mass and obesity associated protein (FTO). J Med Chem.

[81] Martinez S., Hausinger R.P. (2015). Catalytic mechanisms of Fe(II)- and 2-oxoglutarate-dependent oxygenases. J Biol Chem.

[82] Avgustinova A., Benitah S.A. (2016). Epigenetic control of adult stem cell function. Nat Rev Mol Cell Biol.

[83] Wu X., Zhang Y. (2017). TET-mediated active DNA demethylation: mechanism, function and beyond. Nat Rev Genet.

[84] Fu Y., He C. (2012). Nucleic acid modifications with epigenetic significance. Curr Opin Chem Biol.

[85] Aik W., McDonough M.A., Thalhammer A., Chowdhury R., Schofield C.J. (2012). Role of the jelly-roll fold in substrate binding by 2-oxoglutarate oxygenases. Curr Opin Struct Biol.

[86] Chen W., Zhang L., Zheng G., Fu Y., Ji Q., Liu F. (2014). Crystal structure of the RNA demethylase ALKBH5 from zebrafish. FEBS Lett.

[87] Jia G., Yang C.G., Yang S., Jian X., Yi C., Zhou Z. (2008). Oxidative demethylation of 3-methylthymine and 3-methyluracil in single-stranded DNA and RNA by mouse and human FTO. FEBS Lett.

[88] Mauer J., Luo X., Blanjoie A., Jiao X., Grozhik A.V., Patil D.P. (2017). Reversible methylation of m(6)Am in the 5' cap controls mRNA stability. Nature.

[89] Zhang S., Zhao B.S., Zhou A., Lin K., Zheng S., Lu Z. (2017). m(6)A demethylase ALKBH5 maintains tumorigenicity of glioblastoma stem-like cells by sustaining FOXM1 expression and cell proliferation program. Cancer Cell.

[90] Gulati P., Avezov E., Ma M., Antrobus R., Lehner P., O'Rahilly S. (2014). Fat mass and obesity-related (FTO) shuttles between the nucleus and cytoplasm. Biosci Rep.

[91] Su R., Dong L., Li C., Nachtergaele S., Wunderlich M., Qing Y. (2018). R-2HG exhibits anti-tumor activity by targeting FTO/m(6)A/MYC/CEBPA signaling. Cell.

[92] Gulati P., Cheung M.K., Antrobus R., Church C.D., Harding H.P., Tung Y.C. (2013). Role for the obesity-related *FTO* gene in the cellular sensing of amino acids. Proc Natl Acad Sci U S A.

[93] Li Z., Weng H., Su R., Weng X., Zuo Z., Li C. (2017). FTO plays an oncogenic role in acute myeloid leukemia as a *N*(6)-methyladenosine RNA demethylase. Cancer Cell.

[94] Xu C., Liu K., Tempel W., Demetriades M., Aik W., Schofield C.J. (2014). Structures of human ALKBH5 demethylase reveal a unique binding mode for specific single-stranded *N*^6^-methyladenosine RNA demethylation. J Biol Chem.

[95] Feng C., Liu Y., Wang G., Deng Z., Zhang Q., Wu W. (2014). Crystal structures of the human RNA demethylase Alkbh5 reveal basis for substrate recognition. J Biol Chem.

[96] Yang C.G., Yi C., Duguid E.M., Sullivan C.T., Jian X., Rice P.A. (2008). Crystal structures of DNA/RNA repair enzymes AlkB and ABH2 bound to dsDNA. Nature.

[97] Huang Y., Yan J., Li Q., Li J., Gong S., Zhou H. (2015). Meclofenamic acid selectively inhibits FTO demethylation of m^6^A over ALKBH5. Nucleic Acids Res.

[98] Wang T., Hong T., Huang Y., Su H., Wu F., Chen Y. (2015). Fluorescein derivatives as bifunctional molecules for the simultaneous inhibiting and labeling of FTO protein. J Am Chem Soc.

[99] Gerken T., Girard C.A., Tung Y.C., Webby C.J., Saudek V., Hewitson K.S. (2007). The obesity-associated FTO gene encodes a 2-oxoglutarate-dependent nucleic acid demethylase. Science.

[100] Chen B., Ye F., Yu L., Jia G., Huang X., Zhang X. (2012). Development of cell-active *N*^6^-methyladenosine RNA demethylase FTO inhibitor. J Am Chem Soc.

[101] Li Q., Huang Y., Liu X., Gan J., Chen H., Yang C.G. (2016). Rhein inhibits AlkB repair enzymes and sensitizes cells to methylated DNA damage. J Biol Chem.

[102] Flanagan J.U., Yosaatmadja Y., Teague R.M., Chai M.Z., Turnbull A.P., Squire C.J. (2012). Crystal structures of three classes of non-steroidal anti-inflammatory drugs in complex with aldo-keto reductase 1C3. PLoS One.

[103] Wojtas M.N., Pandey R.R., Mendel M., Homolka D., Sachidanandam R., Pillai R.S. (2017). Regulation of m(6)A transcripts by the 3'–>5' RNA helicase YTHDC2 is essential for a successful meiotic program in the mammalian germline. Mol Cell.

[104] Wang X., Lu Z., Gomez A., Hon G.C., Yue Y., Han D. (2014). *N*^6^-methyladenosine-dependent regulation of messenger RNA stability. Nature.

[105] Xu C., Wang X., Liu K., Roundtree I.A., Tempel W., Li Y. (2014). Structural basis for selective binding of m^6^A RNA by the YTHDC1 YTH domain. Nat Chem Biol.

[106] Xiao W., Adhikari S., Dahal U., Chen Y.S., Hao Y.J., Sun B.F. (2016). Nuclear m(6)A reader YTHDC1 regulates mRNA splicing. Mol Cell.

[107] Roundtree I.A., He C. (2016). Nuclear m(6)A reader YTHDC1 regulates mRNA splicing. Trends Genet.

[108] Roundtree I.A., Luo G.Z., Zhang Z., Wang X., Zhou T., Cui Y. (2017). YTHDC1 mediates nuclear export of *N*(6)-methyladenosine methylated mRNAs. Elife.

[109] Ivanova I., Much C., Di Giacomo M., Azzi C., Morgan M., Moreira P.N. (2017). The RNA m(6)A reader YTHDF2 is essential for the post-transcriptional regulation of the maternal transcriptome and oocyte competence. Mol Cell.

[110] Li A., Chen Y.S., Ping X.L., Yang X., Xiao W., Yang Y. (2017). Cytoplasmic m(6)A reader YTHDF3 promotes mRNA translation. Cell Res.

[111] Shi H., Wang X., Lu Z., Zhao B.S., Ma H., Hsu P.J. (2017). YTHDF3 facilitates translation and decay of *N*(6)-methyladenosine-modified RNA. Cell Res.

[112] Du H., Zhao Y., He J., Zhang Y., Xi H., Liu M. (2016). YTHDF2 destabilizes m(6)A-containing RNA through direct recruitment of the CCR4-NOT deadenylase complex. Nat Commun.

[113] Luo S., Tong L. (2014). Molecular basis for the recognition of methylated adenines in RNA by the eukaryotic YTH domain. Proc Natl Acad Sci U S A.

[114] Theler D., Dominguez C., Blatter M., Boudet J., Allain F.H. (2014). Solution structure of the YTH domain in complex with *N*^6^-methyladenosine RNA: a reader of methylated RNA. Nucleic Acids Res.

[115] Zhu T., Roundtree I.A., Wang P., Wang X., Wang L., Sun C. (2014). Crystal structure of the YTH domain of YTHDF2 reveals mechanism for recognition of *N*^6^-methyladenosine. Cell Res.

[116] Li F., Zhao D., Wu J., Shi Y. (2014). Structure of the YTH domain of human YTHDF2 in complex with an m(6)A mononucleotide reveals an aromatic cage for m(6)A recognition. Cell Res.

[117] Xu C., Liu K., Ahmed H., Loppnau P., Schapira M., Min J. (2015). Structural basis for the discriminative recognition of *N*^6^-methyladenosine RNA by the human YT521-B homology domain family of proteins. J Biol Chem.

[118] Bertonati C., Punta M., Fischer M., Yachdav G., Forouhar F., Zhou W. (2009). Structural genomics reveals EVE as a new ASCH/PUA-related domain. Proteins.

[119] Cerrudo C.S., Ghiringhelli P.D., Gomez D.E. (2014). Protein universe containing a PUA RNA-binding domain. FEBS J.

[120] Yamashita A., Takayama T., Iwata R., Yamamoto M. (2013). A novel factor Iss10 regulates Mmi1-mediated selective elimination of meiotic transcripts. Nucleic Acids Res.

[121] Stowell J.A.W., Webster M.W., Kogel A., Wolf J., Shelley K.L., Passmore L.A. (2016). Reconstitution of targeted deadenylation by the Ccr4-Not complex and the YTH domain protein Mmi1. Cell Rep.

[122] Wang C., Zhu Y., Bao H., Jiang Y., Xu C., Wu J. (2016). A novel RNA-binding mode of the YTH domain reveals the mechanism for recognition of determinant of selective removal by Mmi1. Nucleic Acids Res.

[123] Wu B., Xu J., Su S., Liu H., Gan J., Ma J. (2017). Structural insights into the specific recognition of DSR by the YTH domain containing protein Mmi1. Biochem Biophys Res Commun.

[124] Darnell R.B., Ke S., Darnell J.E. (2018). Pre-mRNA processing includes *N*(6) methylation of adenosine residues that are retained in mRNA exons and the fallacy of “RNA epigenetics”. RNA.

[125] Zhao B.S., Nachtergaele S., Roundtree I.A., He C. (2018). Our views of dynamic *N*(6)-methyladenosine RNA methylation. RNA.

[126] Huang J., Dong X., Gong Z., Qin L.Y., Yang S., Zhu Y.L. (2018). Solution structure of the RNA recognition domain of METTL3-METTL14 *N*(6)-methyladenosine methyltransferase. Protein Cell.

[127] Pendleton K.E., Chen B., Liu K., Hunter O.V., Xie Y., Tu B.P. (2017). The U6 snRNA m(6)A methyltransferase METTL16 regulates SAM synthetase intron retention. Cell.

[128] Warda A.S., Kretschmer J., Hackert P., Lenz C., Urlaub H., Hobartner C. (2017). Human METTL16 is a *N*(6)-methyladenosine (m(6)A) methyltransferase that targets pre-mRNAs and various non-coding RNAs. EMBO Rep.

[129] Zou S., Toh J.D., Wong K.H., Gao Y.G., Hong W., Woon E.C. (2016). *N*(6)-methyladenosine: a conformational marker that regulates the substrate specificity of human demethylases FTO and ALKBH5. Sci Rep.

[130] Wei C., Gershowitz A., Moss B. (1975). *N*^6^, ^O^2'-dimethyladenosine a novel methylated ribonucleoside next to the 5' terminal of animal cell and virus mRNAs. Nature.

[131] Linder B., Grozhik A.V., Olarerin-George A.O., Meydan C., Mason C.E., Jaffrey S.R. (2015). Single-nucleotide-resolution mapping of m^6^A and m^6^A_m_ throughout the transcriptome. Nat Methods.

[132] Edupuganti R.R., Geiger S., Lindeboom R.G.H., Shi H., Hsu P.J., Lu Z. (2017). *N*(6)-methyladenosine (m(6)A) recruits and repels proteins to regulate mRNA homeostasis. Nat Struct Mol Biol.

[133] Meyer K.D., Patil D.P., Zhou J., Zinoviev A., Skabkin M.A., Elemento O. (2015). 5′ UTR m(6)A promotes cap-independent translation. Cell.

[134] Huang H., Weng H., Sun W., Qin X., Shi H., Wu H. (2018). Recognition of RNA *N*(6)-methyladenosine by IGF2BP proteins enhances mRNA stability and translation. Nat Cell Biol.

[135] Alarcon C.R., Goodarzi H., Lee H., Liu X., Tavazoie S., Tavazoie S.F. (2015). HNRNPA2B1 is a mediator of m(6)A-dependent nuclear RNA processing events. Cell.

[136] Wu B., Su S., Patil D.P., Liu H., Gan J., Jaffrey S.R. (2018). Molecular basis for the specific and multivariant recognitions of RNA substrates by human hnRNP A2/B1. Nat Commun.

